# MADS-Box Transcription Factor *ZtRlm1* Is Responsible for Virulence and Development of the Fungal Wheat Pathogen *Zymoseptoria tritici*

**DOI:** 10.3389/fmicb.2020.01976

**Published:** 2020-08-18

**Authors:** Naser Mohammadi, Rahim Mehrabi, Amir Mirzadi Gohari, Mozaffar Roostaei, Ebrahim Mohammadi Goltapeh, Naser Safaie, Gert H. J. Kema

**Affiliations:** ^1^Dryland Agricultural Research Institute, Agricultural Research, Education and Extension Organization (ARREO), Maragheh, Iran; ^2^Department of Biotechnology, College of Agriculture, Isfahan University of Technology, Isfahan, Iran; ^3^Department of Plant Pathology, Faculty of Agricultural Sciences and Engineering, College of Agriculture and Natural Resources, University of Tehran, Karaj, Iran; ^4^Department of Plant Pathology, Faculty of Agriculture, Tarbiat Modares University, Tehran, Iran; ^5^Wageningen University and Research, Wageningen Plant Research, Wageningen, Netherlands

**Keywords:** *Zymoseptoria tritici*, gene deletion, pathogenicity assay, phenotyping, *Agrobacterium* tumefaciens-mediated transformation

## Abstract

*Zymoseptoria tritici* is one of the most economically destructive wheat diseases all over the world and is a model fungal plant pathogen within the ascomycetes. In this study, the instrumental role of the *ZtRlm1* gene encoding a MADS-box transcription factor (TF) in the infection process of *Z. tritici* was functionally characterized as these proteins play critical roles in the global gene regulation required for various developmental and physiological processes. Our infection assays showed that *ZtRlm1* mutants were attenuated in disease development as a 30 and 90% reduction in chloro-necrotic lesions and pycnidia formation, respectively, were observed in plants inoculated with *ZtRlm1* mutant strains demonstrating that *ZtRlm1* is a crucial factor playing a significant role in the late stage of infection corresponding with pycnidial formation. Our expression analysis demonstrated that the transcript level of *ZtRlm1* is induced at 2 and 20 days post-inoculation, coinciding with pycnidial sporulation. In addition, microscopic analyses showed that branch intensity and biomass production were significantly reduced, indicating that impaired pycnidia formation is a result of impaired differentiation and biomass production in the *ZtRlm1* mutants. Furthermore, melanization, a phenomenon required for fruiting body formation, was significantly hampered in *ZtRlm1* mutants as they were not melanized under all tested temperature and media conditions. Overall, our data showed that impaired disease development of the *ZtRlm1* mutants is mainly due to the significant impact of *ZtRlm1* in different cellular processes, including differentiation, branching, fungal biomass production, and melanization, in which identification of downstream genes are of interest to increase our understanding of this pathosystem.

## Introduction

*Zymoseptoria tritici* (Desm.) Quaedvlieg & Crous (Quaedvlieg et al., 2011) (formerly known as *Mycosphaerella graminicola*), causes septoria tritici blotch (STB), which is one of the most economically destructive wheat diseases all over the worlds. STB results in imposing remarkable yield losses annually in wheat-growing regions with high rainfall and moderate temperature during the growing season (Eyal and Levy, 1987; Fones and Gurr, 2015). Currently, the application of resistant cultivars is the most cost-effective and environmentally friendly approach to manage STB disease (Brown et al., 2015). However, only a limited number of resistant cultivars are currently commercially applied to manage this damaging disease (Fraaije et al., 2005; Orton et al., 2011). The importance of STB is increasing, particularly after the introduction of high yielding cultivars that are specifically improved for resistance to other biotic and abiotic stresses. In addition, the rapid emergence of fungicide resistant isolates in natural populations of this fungus has brought a strong demand for wheat protection against STB.

*Z. tritici*-wheat pathosystem has attracted considerable attention from many researchers as this fungus has a unique lifestyle, active sexual cycle, genomic feature, and high genome plasticity (Dean et al., 2012). *Z. tritici* is considered as a hemibiotrophic pathogen with a symptomless biotrophic stage of around 10-days, followed by a rapid switch to the necrotrophic stage. The fungus directly penetrates the leaves through natural opening (stomata) by forming appressorium-like swellings at the hyphal tips and afterward intercellularly colonizes the apoplastic space, without establishing particular feeding structures such as haustoria. During the biotrophy, the fungal biomass is low and increases exponentially after switching to necrotrophy in which the typical symptoms of the disease including irregular chlorotic lesions develops leading to the formation of necrotic blotches that eventually bear pycnidia and pycnidiospores in sub-stomatal cavities (Eyal et al., 1987; Kema et al., 1996; Mehrabi et al., 2006a). Sexual reproduction of this fungus can be completed several times during growing seasons resulting in developing natural populations with high genetic diversity (Chen and McDonald, 1996; Hunter et al., 1999; Morais et al., 2019).

To initiate infection, plant pathogenic fungi are able to perceive host signals followed by proper molecular and phenotypic responses, including penetration and colonization. During co-evolution, both pathogens and their hosts have achieved elaborated tactics leading to either compatible or incompatible interactions (Dodds and Rathjen, 2010). For example, fungal pathogens use specific virulence factors to suppress or manipulate host defense mechanisms, whereas host plants have developed molecular components to recognize and activate other defense processes against invading pathogens (De Wit et al., 2009). Plant-pathogenic fungi are equipped with numerous genes to effectively regulate the distinctive developmental and differentiation processes to attack host cells. Additionally, it is well documented that dynamic and complex interactions presented in the battlefield between a host and a fungal pathogen would determine the outcome of the interactions (compatibility/incompatibility). Targeted gene replacement strategies were employed to functionally characterize some of these genes (Mehrabi and Kema, 2006; Mehrabi et al., 2006a, b, 2007, 2009; Orton et al., 2011). Among the genes involved in the infection process, transcription factors (TFs) play pivotal roles in the global regulation of other genes required for various developmental processes as well as a successful infection. For example Mirzadi Gohari et al. (2014) demonstrated that *ZtWor1* was implicated in the infection process of *Z. tritici* since deletion mutant failed to cause disease and complementation of the mutant by *ZtWor1* gene restored the wild-type phenotypes. In another study Mohammadi et al. (2017) showed that *ZtVf1* was involved in different developmental stages, including melanization, hyphal branching, pycnidia differentiation and biomass production and acts as a functional pathogenicity factor. The MADS-box proteins belong to the family of TFs that directly bind to specific motifs in the promoter region of the targeted genes and participate in a diverse range of biological activities (Messenguy and Dubois, 2003). Typically, filamentous ascomycetes have only two MADS-box transcription factor proteins, serum response factor (SRF-type or type I) and myocyte enhancer factor (MEF-type or type II). Unlike many fungi, *Saccharomyces cerevisiae* has four MADS-box transcription factors; Mcm1 and Arg80 proteins are associated with the SRF-type while Rlm1 and Smp1 belong to the MEF-type (de Nadal et al., 2003). In *S. cerevisiae*, Rlm1 is involved in the expression of genes that are essential for cell wall integrity, whereas Smp1 is associated with regulating genes that are responsible for osmotic stress response (de Nadal et al., 2003). Furthermore, Mcm1 and Arg80 regulate arginine metabolism in yeast cells (Messenguy and Dubois, 2003). Targeted gene deletion of *MIG1* (MADS-box protein responsible for infectious growth 1 gene) as homolog of *ZtRlm1* in the fungal plant pathogen *Magnaporthe oryzae* revealed their involvement in plant infection (Mehrabi et al., 2008). In *mig1* mutant strains, infectious structures, including appressoria, penetration pegs, and primary infectious hyphae were generated. However, these mutants were unable to create secondary infectious hyphae in living cells. Additionally, mutant strains of *mig1* were able to penetrate and develop infectious hypha-like structures in plant cells killed by heating or cellophane membranes, indicating that this gene might be acting as a downstream of *Magnaporthe grisea* MAP kinase (*MPS1*) to regulate genes required for suppressing plant defense responses (Mehrabi et al., 2008).

Although few reports showed the instrumental roles of MADS-box TFs in the virulence of various fungal pathogens (Mehrabi et al., 2008; Lin et al., 2018), no functional study has been performed to unveil the functional role of these TFs in *Z. tritici*. Therefore, we functionally characterized the biological function of the *ZtRlm1*, homolog of *MIG1*, in *Z. tritici*, and our results confirmed that this gene is implicated in several developmental processes to successfully establish tissue colonization.

## Materials and Methods

### Biological Materials

We applied *Z. tritici* IPO323, which is highly pathogenic on the wheat cultivar Obelisk (Mehrabi et al., 2006a) in our assays. This strain was propagated on PDA media (Sigma-Aldrich Chemie, Steinheim, Germany) or Yeast Glucose Broth (YGB) medium (yeast extract 10 g/L and glucose 20 g/L) to generate abundant yeast-like cells at 18°C for 5–7 days. Eventually, the cells generated on the cultured plates were harvested by softly scratching cultures, and kept at –80°C (Kema and van Silfhout, 1997).

### Constructs Generation

The gene deletion construct designated as p*ZtRlm1*KO was generated by using the USER-friendly cloning approach to delete the *ZtRlm1* (Frandsen et al., 2008). Firstly, primer pairs, including ZtRlm1-PRF-F1, R1, and ZtRlm1-PRF-F2, R2 to amplify 1.8 kb bp upstream and downstream regions of the targeted genes through *PfuTurbo*^®^ CxHotstart DNA polymerase were applied (Stratagene, Cedar Creek, TX, United States). Additionally, the pRF-HU2 vector contained the hygromycin phosphotransferase (*hph*) gene as a selectable marker was digested via enzymes named *Pac*I and *Nt.Bbv*CI, to create an overhang ends that are compatible with the PCR products. Afterward, the resultant materials were combined and treated with the USER enzyme mix (New England Biolabs, Ipswich, MA, United States) and placed at 37°C for 30 min followed by another incubation period at 25°C for 30 min. Eventually, the developed reaction was directly transformed into *Escherichia coli* strain DH5α using an electroporation machine. Subsequently, the treated materials were cultured on a medium possessing the antibiotic kanamycin. The right bacteria harboring the interesting fragment with the expected insertions at the right places were evaluated using colony PCR technique via *hph*-R2/*ZtRlm1*-PRF-F1 and *hph*-F2/*ZtRlm1*-PRF-R2 primer pairs (Table 1 and Figure 2A). The correct construct amplified 2065 bp bands with both primer pairs, indicating that the upstream and downstream of *ZtRlm1* placed in the right position in the pRF-HU2 vector leading to the development of p*ZtRlm1*KO. All primers are listed in Table 1.

**TABLE 1:** Primers used in this study.

Primer name	Sequence (5’–3’)
ZtRlm1-PRF-F1	GAGGGCAATGTGTTCAGACTGG
ZtRlm1-PRF-R1	CGACCCGGAACCTGGCCAA
ZtRlm1-PRF-F2	GGACTTAAUGGTGAGGAGCGAGGGAGG
ZtRlm1-PRF-R2	GGGTTTAAUGGTGAGGACGGAGATTTGGCTT
ZtRlm1-F1	GGGTCGTCGAAAGATTGAGATCAAA
ZtRlm1-R1	TGGATTGTGAGCGTGGTCCAA
hph-F2	CAGCCAAGCCCAAAAAGTGCTC
ZtRlm1-R2	TTGCGAGTTGTTGGTCGAGGA
hph-R1	TGGCTTGTATGGAGCAGCAGA
hph-F1	GAAGAGGAGCATGTCAAAGTACAATT

### Fungal Transformation

We cloned the p*ZtRlm1*KO into *Agrobacterium tumefaciens* strain LBA1100 through electroporation technique. *A. tumefaciens-*mediated transformation (ATMT) method was performed to delete *ZtRlm1* in the *Z. tritici* IPO323, as previously described (Mehrabi et al., 2006a). Following 20 days, separate *Z. tritici* transformants were picked up and transferred to a PDA medium supplemented with 100 μg/mL hygromycin plus 200 μg/mL cefatoxime. We subsequently extracted the genomic DNAs of each transformant based on the protocol depicted by the Dellaporta et al. (1983). They extracted DNA were applied in a PCR-based screening approach to test the presence of expected fragments. Our PCR-based screening showed that the band of 743 bp using the pair primer (*ZtRlm1-*F1 and *ZtRlm1-*R1) was amplified in the WT and ectopic transformants, whereas this band was not generated in the deleted mutant for *ZtRlm1* (Figure 2B), implying that this gene was eliminated from these independent transformants. All the transformants including ectopic and *ZtRlm1* mutants amplified a band of 764 bp, when *hph* specific primers (*hph*-F1 and *hph*-R1) were used in PCR amplification (Figure 2C). Additionally, elimination of *ZtRlm1* occurred in the chromosomal region was investigated by a primer pair named *hph-*F2 and *ZtRlm1* -R2 demonstrating the anticipated band of 2065 bp were exclusively amplified in the deleted mutant for *ZtRlm1* (Figure 2D).

### Infection Assay

The wheat cv. Obelisk was grown in a glasshouse by the time that the first leaves have appeared. Inoculum of the tested strains was generated in the YGB at 18°C for 7 days, and yeat-like cells were collected by centrifuging at 3000 rpm for 3 min followed by two washing steps by distilled water. Subsequently, spore concentrations were adjusted to 10^7^∘ml^–1^ spores by a hemocytometer instrument, and the 0.15% Tween 20^®^ was added to the spore suspensions to enhance spore adhering to leaf surfaces. Infection assay was performed as described in detail previously (Mohammadi et al., 2017) in three replicates and each replicate contained a pot of 5 seedlings. The infected plants were kept under black plastic bags for 48 h to increase the humidity and then transferred to a greenhouse compartment at 22°C with a relative humidity > 90% and 16 h light and 8-h darkness. Disease development was evaluated daily and recorded following disease symptoms expression around 8 dpi, which is coincident with the transition phase. We documented the percentage of chloro-necrotic regions along with the chloronecrotic lesions covered by asexual fruiting bodies (pycnidia). At 21 dpi, 10 leaves infected by all examined strains were harvested, and the formed pycnidia in the distance down to 1 cm from the leaf tips were calculated and analyzed using SPSS software package (IBM SPSS Statistics 19, United States).

### RNA Isolation and Quantitative RT-PCR

Transcript abundance of *ZtRlm1* at various time courses was conducted through a quantitative real-time PCR (q-RT-PCR) technique. For evaluating the expression level of *ZtRlm1* i*n planta*, we infected the wheat cv. Obelisk by *Z. tritici* IPO323 and, subsequently, the inoculated leaves were harvested in three biological replicates (four leaves per each biological replicates) (Mehrabi et al., 2006a). The samples were immediately frozen by placing them in the liquid nitrogen and, then, ground in this liquid via a mortar and pestle. Total RNA of the inoculated leaves and fungal biomass generated in YGB was extracted through the RNeasy plant mini kit (Qiagen, location, United States). We applied the DNA-free kit (Ambion, Cambridgeshire, United Kingdom) to eliminate DNA contamination, and afterward synthesized the first-strand cDNA from around two μg of total RNA primed with oligo(dT) via the SuperScript III enzyme based on the instructions provided by the manufacturer. One μl of the synthesized cDNA was employed in a 25 μl PCR reaction via a QuantiTect SYBR Green PCR Kit (Applied Biosystems, Warrington, United Kingdom), and run and analyzed using an ABI 7500 Real-Time PCR machine. Three technical replicates for each biological replicates were used, allowing us to perform the statistical analysis through the SPSS software package (IBM SPSS Statistics 19, United States). We initially normalized the relative expression of *ZtRlm1* with the constitutively expressed *Z. tritici* beta-tubulin gene (Keon et al., 2007; Motteram et al., 2009) and, eventually, estimated according to the comparative C (t) method defined previously (Schmittgen and Livak, 2008).

### *In vitro* Phenotyping

We used two nutritionally diverse solid cultures, including YMDA (4 g yeast, 4 g malt, 10 g dextrose, and 15 g agar in 1 L distilled water) and PDA along with an application of three various temperatures (16, 20, and 28°C) to characterize morphologically the WT and mutant strains. We spotted ~1 μl of spore suspension (10^7^ spores∘ml^–1^) on the above-cultures, and we monitored and recorded their morphological features for 10 days.

We harvested spores of the WT and mutant strains of *ZtRlm1* produced in the YGB medium at 18°C for 5 days. Afterward, we washed spore suspensions by the distilled water to remove remaining media and, subsequently, adjusted the spores to 10^5,^ spores ml^–1^. Next, we placed 12μl of the adjusted spore suspensions on a piece of the Water Agar (WA) or PDA fragment placed on a microscope slide covered by a coverslip. Eventually, we incubated the prepared materials at 20°C under dark conditions, and at least two spores of each sample were analyzed using an Olympus BX51 microscope equipped with Olympus DP72 digital camera every 12 h until 48 h. Pictures were proceeded with Adobe Photoshop version 15.2.2. After 48 h, for comparison of spores biomass and spore branches, randomly 10 spores of mutants and control strains cultivated on the PDA and WA media, were selected and analyzed using Digimizer version 4.1.1.0 (MedCalc, 2009) and SPSS softwares package (IBM SPSS Statistics 19, United States).

### *In vivo* Histopathological Assay

We employed two methodologies to determine the germination and penetration patterns, plus the colonization of *Z. tritici* in the attacked leaves. In the first method, we collected the inoculated leaves at various time courses, including 8, 12, 16, and 20 dpi and then we instantly immersed them in 15 ml of 0.05% trypan blue dissolved in lactophenol-ethanol (1:2, v/v), and boiled for 10 min. We subsequently destained samples in a saturated chloral hydrate solution (5:2, w/v) for at least 10 h and then stored in 87% (v/v) glycerol by the time of analysis. In another method, we cleared the harvested samples with a mixture of glacial acetic acid: absolute ethanol (1:3, v/v) followed by transferring onto filter paper saturated with lactoglycerol (1:1:1, lactic acid: glycerol: water, v/v/v) until analysis by a cytological observation (Shetty et al., 2003).

### Phylogenetic Tree Construction

We employed the MEGA software package version 5.05 (Tamura et al., 2011) to construct and analyze the phylogenetic tree. We applied the unweighted pair group approach with the arithmetic average (UPGMA) algorithm to build up the phylogenetic tree, and we evaluated the accuracy of the constructed phylogenetic tree by running the bootstrap analysis of 1000 repetitions.

## Results

### Identification and Characterization of *ZtRlm1*

**FIGURE 1:**
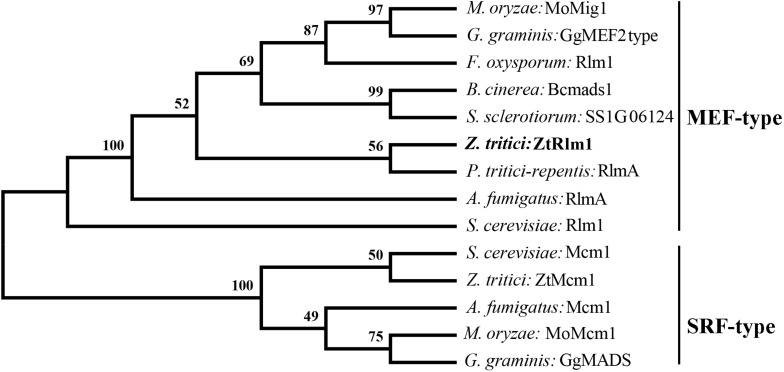
Phylogenetic comparison of *Zymoseptoria tritici* Rlm1 (ZtRlm1) with members of the MADS-box family based on amino acid sequence alignments. The two subfamilies of MADS-box family: SRF-like/Type I and MEF2-like/type II are indicated. The accession number of the proteins are included as ENH69552.1 (Rlm1) (*Fusarium oxysporum*), XP_001559829.1 (Bcmads1) (*Botrytis cinerea*), XP_001593202.1 (SS1G06124) (*Sclerotinia sclerotiorum*), XP_001938412.1 (RlmA) (*Pyrenophora tritici-repentis*), NP_013757.1 (Mcm1) and NP_009741.3 (Rlm1) (*Saccharomyces cervisiae*), XP_003852780.1 (ZtMcm1) and XP_003853236.1 (ZtRlm1) (*Zymoseporia ritici*), XP_747866.1 (Mcm1) and XP_754763.1 (RlmA) (*Aspergillus fumigatus*), XP_003720973.1 (MoMcm1) and XP_003714096.1 (MoMig1) (*Magnaporthe oryzae*), XP_009220065.1 (GgMADS) and XP_009220065.1 (GgMEF2 type) (*Geaumaomyces graminis*). The phylogenetic tree was constructed using MEGA 5 software. The bootstrap values (1000 replicates) are shown above the branches.

To identify the homolog of Rlm1 in the fully sequenced genome of *Z. tritici*, a BLASTp search of *Z. tritici* genome using *S. cerevisiae* Rlm1 (GenBank number: BAA09658.1) as the query was performed resulting in the retrieval of the homolog of Rlm1 protein (protein ID = 71585). This protein was designated as *ZtRlm1* and subsequently subjected to further analyses. *ZtRlm1* has an open reading frame of 1903 bp, which is interrupted by three introns, and it is located on chromosome 4 encoding a protein of 597 amino acids. Interestingly, the overall similarity and identity between *ZtRlm1* and yeast *Rlm1* is about 34%, but they are over 90% identical in the MADS-box domain. Phylogenetic analysis of *ZtRlm1* and related MADS-box proteins from other selected fungi indicated ZtRlm1 grouped with MEF-type MADS-box proteins (Figure 1). The specific motif, including the 58-amino-acid MADS-box region and 75-amino-acid MEF2 region determining the features of TFs are located at the N terminus region of ZtRlm1. Alignment analysis showed that that the N terminus region of *ZtRlm1* is more conserved than the other part of protein sequences.

### Deletion of *ZtRlm1*

**FIGURE 2:**
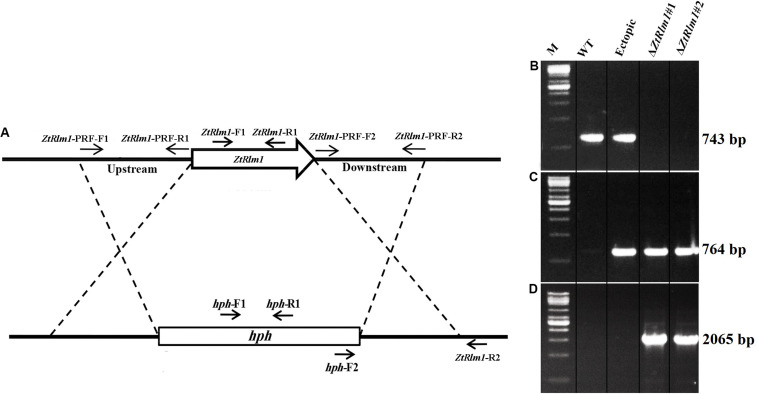
Generation of the *ZtRlm1* gene replacement mutant. Diagram showing the replacement of *ZtRlm1* by hygromycin phosphotransferase resistance cassette (*hph*) through homologous recombination. The dotted lines depict the flanking regions where homologous recombination occurred **(A)**. Identification of replacement mutants by PCR approach. The WT and ectopic transformants generated the expected PCR band (743 bp) using primers *ZtRlm -*F1 and *ZtRlm1 -*R1, while this band was not amplified in the *ZtRlm1* mutants indicating that the *ZtRlm1* gene was deleted from these independent transformants **(B)**. The *ZtRlm1* mutants and ectopic transformants amplified the expected hph band (764 bp) using *hph*-F1/*hph*-R1 primers, whereas this band was absent in the WT **(C)**. A primer pair (*hph-*F2 and *ZtRlm1*-R2) was used to confirm targeted gene deletion that occurred in the *ZtRlm1* chromosomal region. Only the mutant strains were able to produce the expected amplicons (2065 bp) **(D)**.

To study the biological role of *ZtRlm1* during the infection process of *Z. tritici*, the deletion construct based on the USER-friendly method as described previously (Frandsen et al., 2008) was made. As shown in Figure 2A, about 1.8 kb of upstream and downstream of the *ZtRlm1* were used to generate the deletion construct that eventually was used to knock-out the *Z. tritici ZtRlm1* in IPO323 strain through *Agrobacterium tumefaciens–*mediated transformation (ATMT). After transformation, the *ZtRlm1* mutants were found using the *ZtRlm1* gene-specific primers, *ZtRlm1*-F1, and *ZtRlm1* -R1 (Figures 2A,B). After performing many transformations and screening more than 600 transformants, two independent mutants were identified and designated as Δ*ZtRlm1#*1 and Δ*ZtRlm1#*2. *ZtRlm1* gene-specific primers confirmed that only the WT and ectopic transformants could produce a band of 743 bp while this PCR band was absent in the *ZtRlm1* mutants showing that the *ZtRlm1* was deleted in these mutants (Figure 2B). In addition, the *ZtRlm1* mutants and ectopic transformants amplified the expected hph band (764 bp) whereas this band was absent in the WT showing that the construct was inserted in the genome of transformants (Figure 2C). The exact position of homologous recombination in the *ZtRlm1* chromosomal region was also confirmed using the *hph*-F2 primer located at 3’ end of the *hph* marker gene along with the *ZtRlm1* -R2 primer located 211 bp at the right border of *ZtRlm1* downstream (Figure 2D). The results revealed that only *ZtRlm1* mutants could amplify the expected band of 2065 bp, showing that the *ZtRlm1* was exclusively deleted in the mutant strains (Figure 2D). Eventually, it worth mentioning that our laboring effort to generate a complemented strain of Rlm1 deletion mutant was unsuccessful. After transformation, we have screened more than 400 transformants, but none of them were positive.

### *ZtRlm1* Delayed Disease Development

**FIGURE 3:**
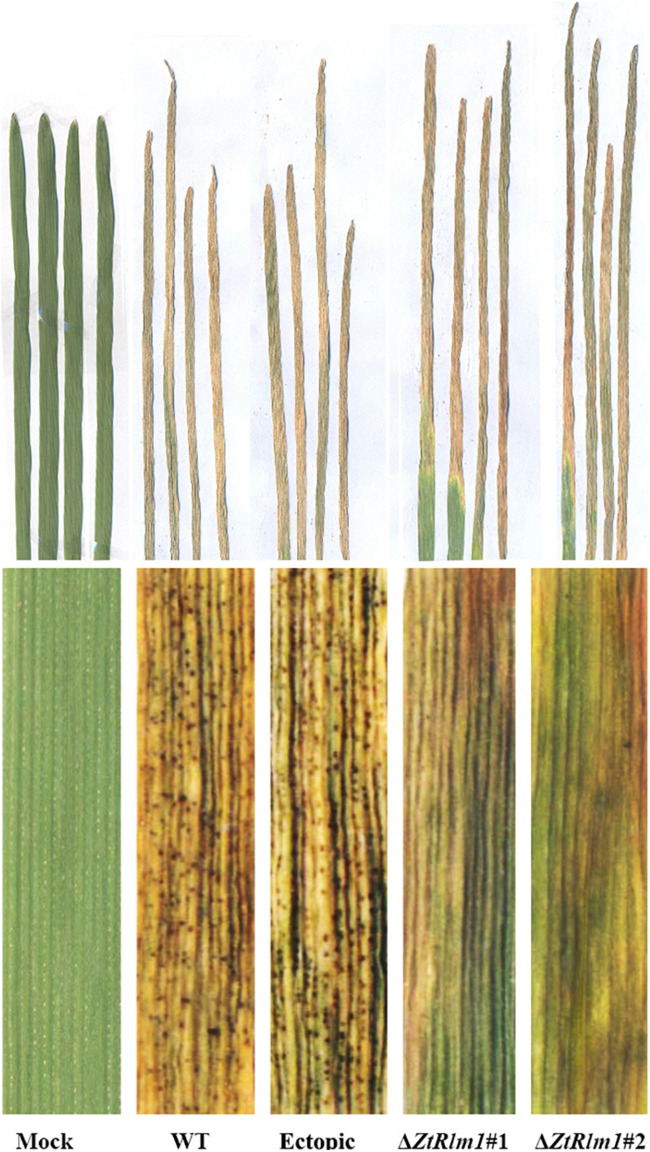
Determination of the effect of *ZtRlm1* deletion on disease development of *Zymoseptoria tritici* on the susceptible wheat cv. Obelisk at 20 dpi. Note that the WT and ectopic strains caused extensive necrosis bearing abundant pycnidia, while the *ZtRlm1* mutant strains generated significantly reduced necrotic regions without pycnidia.

**FIGURE 4:**
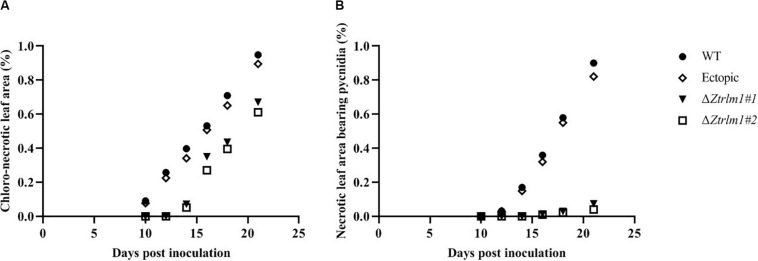
Disease development of *Zymoseptoria tritici ZtRlm1* mutant strains compared with that of the control strains over a period of 10 days (from 10 to 20 days post-inoculation). The average of chloro-necrotic leaf area (%) **(A)**. The average of necrotic leaf area bearing pycnidia **(B)**. Note that the disease development of the *ZtRlm1* mutant strains was significantly reduced compared with that of the control strains.

Infection assay was performed by the inoculation of susceptible wheat cv. Obelisk using the mutant strains (Δ*ZtRlm1#*1 and Δ*ZtRlm1#*2) and the control strains (IPO323 wild type (WT) and ectopic transformants) and disease development was monitored every 48 h. Both WT and ectopic strains caused chlorotic flecks at 9 days post-inoculation (dpi), especially at the leaf tips, which generated extended chlorosis at 12 dpi, and eventually expanded into large necrotic areas containing abundant mature pycnidia at 16–18 dpi (Figure 3). In contrast, *ZtRlm1* mutants were not able to generate disease symptoms until 13 dpi when a few scattered chlorotic and necrotic lesions were observed that slowly merged into larger lesions along with the expansion of chlorotic areas becoming necrotic at 20 dpi but did not sporulate pycnidia even at 30 dpi. The results showed that both independent mutants were unable to produce pycnidia and significantly reduced the percentage of chloro-necrotic lesions (Figure 4 and Supplementary Figure 1). In order to determine the effect of *ZtRlm1* deletion on chlorosis and pycnidia formation, two disease indices (CN and P) were used. In contrast to control strains, the *ZtRlm1* mutant strains did not generate symptoms until 13 dpi when limited chlorotic areas developed that progressively merged into necrotic at 20 dpi without mature and normal pycnidial sporulation (Figures 3, 4). CN and P analysis revealed a significant reduction in CN (~30%) and P (~90%) in plants inoculated with *ZtRlm1* mutant strains compared to that of the WT strain, indicating that *ZtRlm1* is vital for the pycnidial formation (Figure 4 and Supplementary Tables 1–3).

### *ZtRlm1* Plays in Early and Late Phases of Infection

**FIGURE 5:**
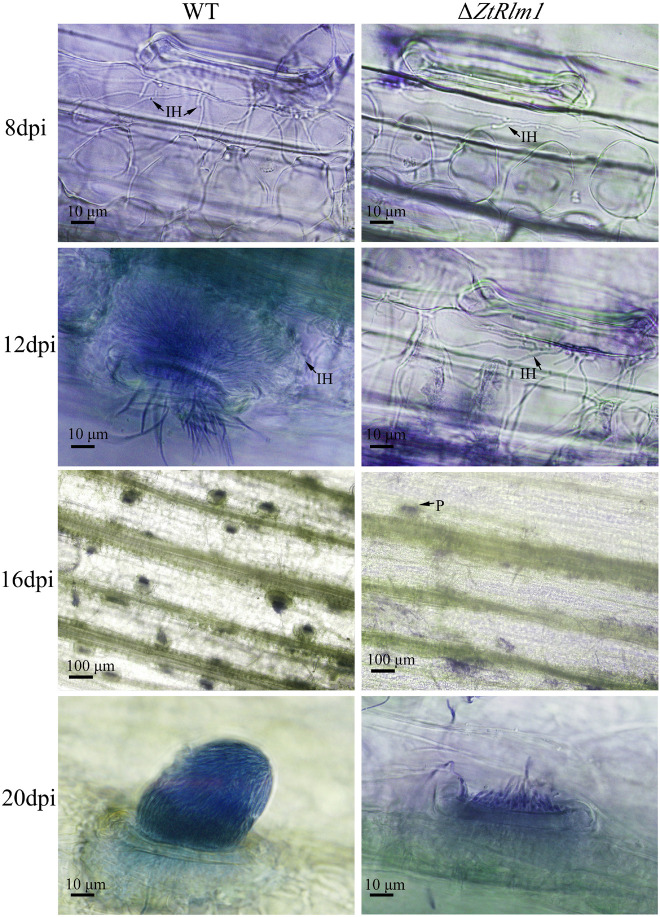
Comparative histopathological analysis of *Zymoseptoria tritici* WT and *ZtRlm1* mutant strain on the susceptible wheat cv. Obelisk at 8, 12, 16, and 20 days post-inoculation (dpi). At 8 dpi, the colonization of mesophyll cells by infectious hypha (IH) of the *ZtRlm1* mutant strain was significantly less than the WT strain. At 12 dpi, the colonization intensity of stomatal cavities in the *ZtRlm1* mutant was significantly lower than that of the WT strain. At 16 and 20 dpi, the infected stomata by the WT resulted in mature pycnidia (P), while in the *ZtRlm1* mutant strain, the majority of infected stomata produced immature pycnidia.

As infection assay indicated that *ZtRlm1* mutants were significantly impaired in virulence, we performed a histopathological study to determine and monitor the behavior of Δ*ZtRlm1* strain during infection stages in further detail. The WT and ectopic strains were able to penetrate stomata by infectious hypha, whereas Δ*ZtRlm1* strain demonstrated a 30% reduction in penetration frequency compared with that of control strains (Supplementary Table 1). Additionally, the *ZtRlm1* mutant strain, in contrast with WT, could not colonize mesophyll cells completely (Figure 5), suggesting that *ZtRlm1* has a pivotal role in the penetration strength as well as the initial establishment of *Z. tritici* colonization. At 12 dpi, infectious hypha of the WT strain totally colonized substomatal cavities of penetrated stomata which was significantly different from the *ZtRlm1* mutant where ∼65% of the penetrated stomata was colonized (Supplementary Table 1). Additionally, the biomass of infectious hypha was much lesser compared to the WT strain. This finding might be the reason for no disease symptom expression until 13 dpi. Finally, the inoculated plants by the *Z. tritici* IPO323 resulted in the pycnidial sporulation at 16 and 20 dpi while that of the *ZtRlm1* deletion mutant strain was failed to produce mature pycnidia at the mentioned time points and we rarely noticed few immature pycnidia in the infected plants by *ZtRlm1* mutant strain in microscopic analysis (Figure 5). These findings indicated that *ZtRlm1* plays a significant function in both of pycnidial production and differentiation (Supplementary Table 1 and Supplementary Figure 1).

### *ZtRlm1* Is Induced at Early and Late Stages of Infection

**FIGURE 6:**
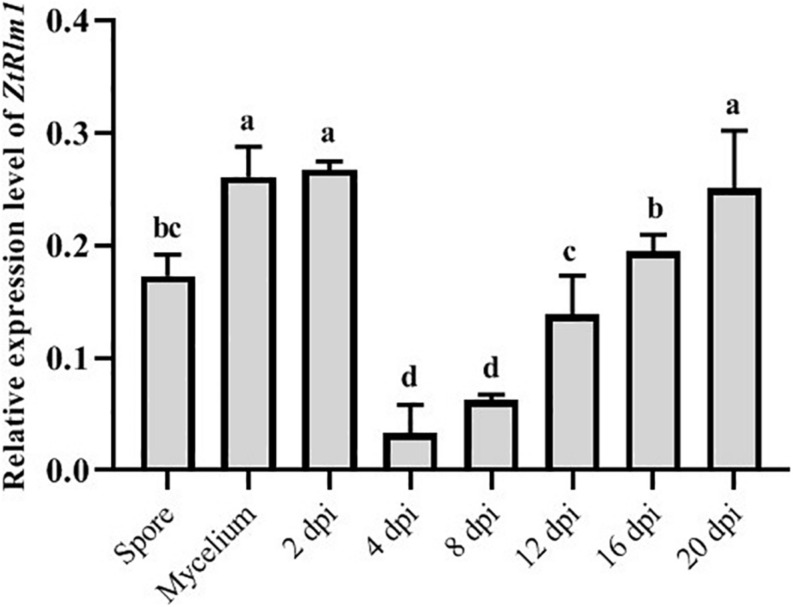
| *In vitro* and *in planta* expression levels of *Zymoseptoria tritici Rlm1*. *In vitro* conditions (18 and 25°C to induce yeast-like cells and mycelia formation, respectively) were selected to compare the expression levels of *ZtRlm1* in mycelial and conidial cultures with *in planta* conditions. The susceptible cv. Obelisk was infected with the *Z. tritici* WT strain and inoculated leaves were collected 2, 4, 8, 12, 16, and 20 days after inoculation followed by RNA isolation and cDNA synthesis. The expression level of *ZtRlm1* was normalized with the constitutively expressed *Z. tritici* beta-tubulin gene. Results display the mean ± SD of three biological reps with three technical replicates for each biological replicates. Bars with different alphabets are significantly different (*p* < 0.05).

As our result showed that *ZtRlm1* acts in the infection process of *Z. tritici*, we analyzed the transcript abundance of *ZtRlm1 in vitro* and *in planta* using a quantitative RT-PCR approach. *ZtRlm1* expression is induced during the early stage of infection (2 dpi), and subsequently, its expression was decreased sharply by 8 dpi. Again, relative expression of *ZtRlm1* remarkably was triggered by 20 dpi, the stage of infection corresponding to the pycnidial formation. The transcript accumulation of *ZtRlm1* in mycelial condition was similar to the *in planta* expression at 2 dpi, whereas that of *ZtRlm1* in yeast-like cells was similar to the *in planta* expression at 12 dpi coinciding with the asexual reproduction (Figure 6).

### *ZtRlm1* Impacts Germination Pattern, Hyphal Branching, and Biomass Production

**FIGURE 7:**
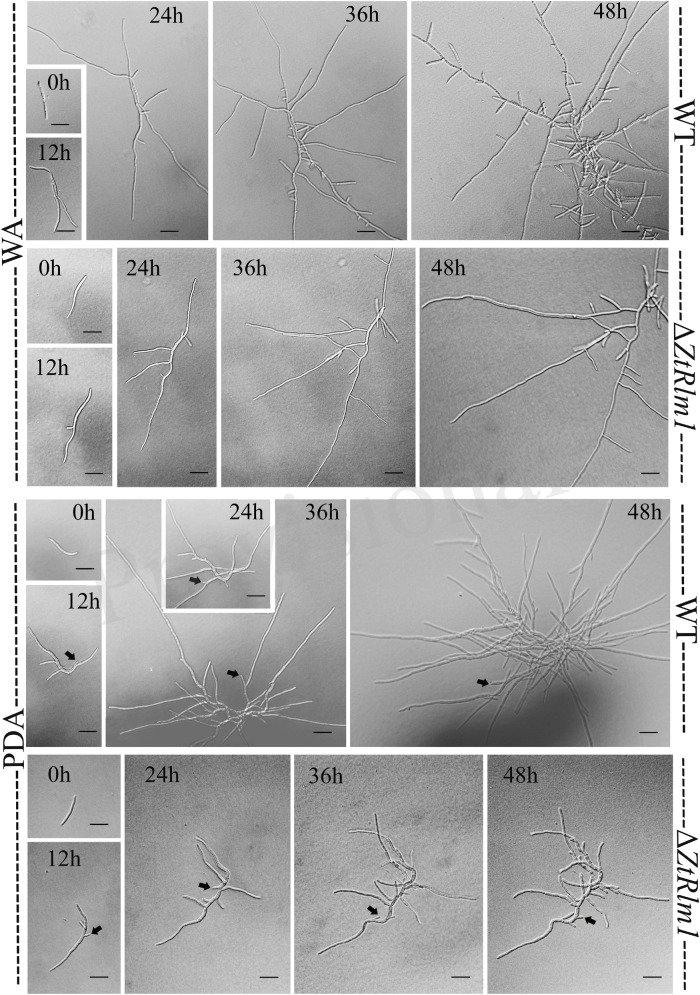
Comparative analysis of the germination pattern of *Zymoseptoria tritici ZtRlm1* mutant compared with that of the WT strain over 48 h. Strains were grown on WA (upper panel) and PDA (lower panel) at 20°C under dark conditions. The branch intensity, as well as filamentation, were reduced in the *ZtRlm1* mutant resulting in less dense filaments compared to the WT strain. The black arrows indicate the budding cells. Bars represent 20 μm.

A successful infection requires proper differentiation and the establishment of a parasitic relationship between the pathogen and the host. Differentiation starts from the development of germ tubes that eventually develop into infectious hyphae to penetrate host tissue. We evaluate the impact of *ZtRlm1* in germination, growth pattern, hyphal branching, and biomass production, by applying two solid media, including water agar (WA) and potato dextrose agar (PDA) and monitor the germination pattern of *ZtRlm1* spores every 12 h (Figure 7). WA is expected to mimic the leaf surface conditions as this induces *Z. tritici* to establish germ tubes as noticed on leaves surface of wheat before penetration (Kema et al., 1996; Mehrabi and Kema, 2006). On WA, we observed that spores of the control strains germinated from both apical cells and formed primary germ tubes within 12 h. The secondary germ tubes established from the same or other cells of the spore after 24 h, which was followed by the expansion of tertiary hyphal as detected within 36 h. Eventually, the tertiary hyphal developed from the primary and secondary germ tubes after 48 h and established the web of compressed filaments (Figure 7). This showed that the poor medium promotes filamentation growth. Furthermore, we did not detect significantly changed germination patterns for the Δ*ZtRlm1* strains compared with that of the control strains after 36 h. Nevertheless, the branch intensity and biomass formation were remarkably decreased to 85 and 60% compared with that of the WT control, respectively (Supplementary Tables 2, 3), culminating in less dense filamentation growth compared with that of the WT strain.

**FIGURE 8:**
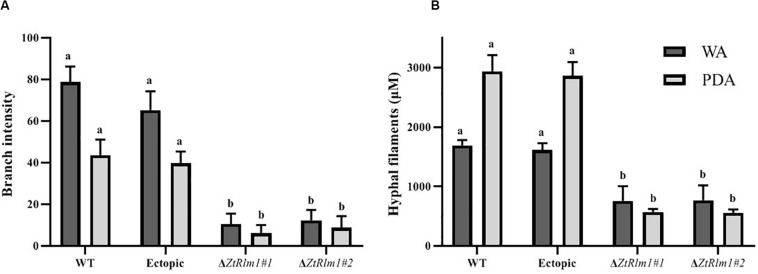
The *ZtRlm1* affects growth and development in *Zymoseptoria tritici*. The WT, ectopic and *ZtRlm1* mutant strains were grown on WA and PDA at 20°C in darkness, and the data was recorded after 48 h. The average branch intensity **(A)** and hyphal filaments **(B)** of *ZtRlm1* mutants were significantly reduced compared with that of the control strains. ^a,b^Statistically significant differences.

The early pattern of colony development on PDA was significantly different in a comparison between the WT and the *ZtRlm1* mutant strain. Following germination event, germ tubes did not extend longitudinally but rather develop simultaneously budding cells on intermediate cells of the spore, and on secondary filaments hyphae during the first 24 h continued by producing intensely extra budding cells partially extended in comparatively short filamentous hyphae at 36 h and stored in the center of the dense colony at 48 h. The germination pattern of *ZtRlm1* mutant strains was remarkably dissimilar from the WT strain. Furthermore, the filamentation event was much limited and delayed filamentous growth in the *ZtRlm1* mutant strain was observed. The hyphae produced by the *ZtRlm1* mutant strain was thicker and high bulbous than those formed WT, and germ tubes turned into unusual hyphal swellings after 48 (Figure 7). We confirmed that the branch intensity and biomass production were significantly and statistically reduced by 83 and 89% in comparison with that of the WT control, respectively (Figure 8 and Supplementary Tables 2, 3).

### *ZtRlm1* Affects Melanization Phenomenon

**FIGURE 9:**
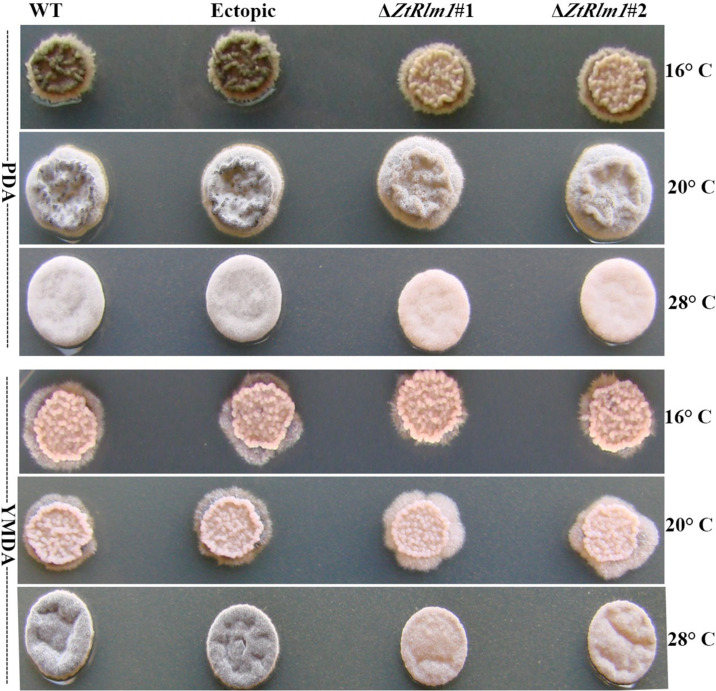
Macroscopic colony morphology of *Zymoseptoria tritici* WT, ectopic, Δ*ZtRlm1 #*1, Δ*ZtRlm1 #*2 strains under different growing conditions. Approximately 2 μl of spore suspension (10^7^ spores/mL) was spotted on PDA (top panel) or YMDA (bottom panel) and grown for 10 days at 16, 20, and 28°C.

We inoculated the WT and ectopic strains along with the Δ*ZtRlm1* strains on two diverse solid media (PDA and YMDA) under three temperature conditions for 10 days to investigate the instrumental role of *ZtRlm1* in melanization event (16, 20, and 28°C). Colony morphology and pattern growth of the Δ*ZtRlm1* strains on applied media at three different temperatures did not show significant differences and were similar to that of the control strains. Interestingly, melanization did not occur under all conditions tested, even after extending an incubation period to 1 month (Figure 9).

## Discussion

To successfully establish a parasitic relationship with its host plant, *Z. tritici* utilizes diverse tactics to colonize its host efficiently. Up to now, some virulence factors like *ZtWor1*, and *ZtVf1*, (Mirzadi Gohari et al., 2014; Mohammadi et al., 2017) have been functionally characterized and to some extent, their contributions to the infection process of *Z. tritici* were investigated to understand how this fungus evade defense mechanisms and establishes STB. Despite this fact that several pathogenicity factors of *Z. tritici* being crucial during different stages of the infection process are functionally characterized (Orton et al., 2011; Mirzadi Gohari et al., 2014), molecular strategies underlying *Z. tritici* infection process remains weakly understood.

Here, we functionally analyzed the biological role of ZtRlm1, and our results revealed that this protein is required for successful infection and an asexual fructification *in planta*. To fully understand the instrumental role of *ZtRlm1* in other developmental processes such as penetration, colonization, and pycnidial formation, a cytological analysis was carried out. Eventually, we demonstrated that *ZtRlm1* is involved in penetration, colonization, pycnidial formation, and differentiation (Figure 5).

The members of MADS-box TFs contain a preserved motif within the DNA binding domains of these proteins (Shore and Sharrocks, 1995), and it was demonstrated several proteins belonging to this family have substantial functional roles in a variety of organisms (Mehrabi et al., 2008; Delgado-Silva et al., 2014). Like other filamentous ascomycetes, *Z. tritici* has only two MADS-box transcription factors (XP_003852780.1 and XP_003853236.1) that we named them as *ZtRlm1* and *ZtMcm1*.

MADS-box TFs possess a wide array of biological functions, and their contributions to diverse developmental processes as well as virulence in fungal pathogens have been functionally investigated. It is shown that Rlm1 homologs in *Aspergillus niger*, *A*. *nidulans*, and *Candida albicans* play critical roles in maintaining the cell wall integrity (CWI) in reacting to cell wall- perturbing agents through transcriptional regulation of the genes encoding proteins associated with cell wall (Fujioka et al., 2007; Delgado-Silva et al., 2014; Rocha et al., 2016). *A*. *fumigatus RlmA* is required for reinforcing the CWI and virulence since mutant strains deleted for *RlmA* showed a changed cell wall arrangement and tolerance to cell wall stress agents. Additionally, the mutant strains had weakened virulence in a neutropenic murine model of invasive pulmonary aspergillosis (Rocha et al., 2016). Furthermore, it was demonstrated that *M. oryzae* Mig1, an ortholog of *S. cerevisiae* Rlm1, is involved in the development of the secondary infectious hyphae inside the living cells of the plant as the deleted strains were blocked in this stage, thereby they were failed to infect rice leaves. Finally, Bcmads1encoding MEF-type MADS-box TF was known to play an instrumental role in the pathogenicity of *Botrytis cinerea* through its impact on the protein secretion process.

Here, we demonstrated that *ZtRlm1* play a significant role in diverse developmental process, and pathogenicity of *Z. tritici*. Our *in vitro* phenotyping assays suggested that *ZtRlm1* is responsible for hyphal branching growth since lowly branched hypha in Δ*ZtRlm1* under both WA and PDA media was observed, indicating that this gene is a positive regulator of both hyphal growth and branching. This might be a reasonable explanation to interpret the results of infection assay, and significantly reducing the number of pycnidial formation on plants inoculated by control strains and Δ*ZtRlm1*.

It is generally hypothesized that controlled branching promotes fungal invasion as previously demonstrated that hyphal branching probably increases surface area for colonization, which is a critical issue for successful infection (Harris, 2008). Additionally, hyphal branching might enable the fusion of hyphae, thereby facilitating the process of exchanging genetic materials between diverse hyphae of the same or distinctive fungi. Additionally, in *Z. tritici*, pycnidial formation is initiated in substomatal cavities by enormous growth, branching and a wide fusion of hyphae deriving originally from 1 or 2 hyphae resulting in exponential improve in fungal biomass generation coinciding with disease symptoms expression (Hilu and Bever, 1957; Kema et al., 1996; Shetty et al., 2003). Several studies have identified TFs such as Tup1 and Rbp1 that are key regulators of filamentious growth (Braun et al., 2000). A mutant strain deleted for *tup1* showed uncontrolled filamentous growth under all tested conditions, suggesting a key role in the filamentation process (Braun and Johnson, 1997; Celera and Claderone, 2001). These studies proved that the successful colonization of plants by invading fungal pathogens necessitates efficient and normal hyphal branching, hyphal fusion and colonization, and defect in one of these processes culminated in reduced virulence in host plants.

We demonstrated that *ZtRlm1* is a crucial factor playing a major role in the late stage of infection coinciding with pycnidial formation. This data is in agreement with that of *in vivo* expression analysis when *ZtRlm1* expression was highly induced at 20 dpi corresponding to the pycnidia sporulation. Furthermore, *in vitro* expression of *ZtRlm1* at mycelial form is comparable with that of *in planta* at 2 dpi when yeast-like cells of *Z. tritici* germinated to produce infectious hyphae just before penetration. Interestingly, we proved that *ZtRlm1* is a pivotal regulator of melanization event in the *Z. tritici* as the examined mutant strains were not melanized under all tested temperature and media conditions. The melanization in *Z. tritici* is a complex process, and several studies previously indicated that different genetic factors partly have roles in melanin biosynthesis (Mehrabi et al., 2006a, b, Mehrabi and Kema, 2006; Cousin et al., 2006; Lendenmann et al., 2014; Derbyshire et al., 2018). Although our observations demonstrated that *ZtRlm1* plays an important function in the melanization phenomenon of the *Z. tritici*, molecular mechanisms underlying this phenotypic observation is unknown. The future in-depth studies are required to further explore the association of the *ZtRlm1* with genes involved in the melanin biosynthesis pathway.

Our previous functional analysis of *ZtPKS* genes encoding polyketide synthases demonstrated that melanization process is not involved in the pathogenicity of *Z. tritici* in contrast to other phytopathogens such as *Magnaporthe oryzae* that has been demonstrated that an appressorium melanization is a central event in the infection process (Casadevall et al., 2000; Jacobson, 2000; Hamilton and Gomez, 2002; Eisenman and Casadevall, 2012). Nevertheless, the melanin is a multifunctional agent, providing defense against environmental stresses but a major role of melanin in plant-pathogenic fungi is the pigment’s contribution to virulence by diminishing the susceptibility of fungi to host defense strategies.

It has been previously shown that the pycnidia of *Z. tritici* are heavily melanized during the infection process (Kema et al., 1996; Duncan and Howard, 2000; Mehrabi and Kema, 2006), suggesting that melanization event is required for asexual fructification. Thus, this may explain the failure of the *ZtRlm1* mutants to produce pycnidia *in vitro*.

To sum up, we conclude *ZtRlm1* is a putative transcriptional regulator in *Z. tritici* playing a central function in the diverse developmental process, including differentiation, asexual fructification, and pathogenicity. As this TF have combinatorial interactions with other regulatory proteins involved in the signaling cascade, it is required to discover the downstream components of *ZtRlm1* that lead to the identification of further pathogenicity factors regulating morpho-pathogenic behavior of *Z. tritici*. This will contribute to the further understanding of the *Z. tritici* – wheat pathosystem.

## Data Availability Statement

The datasets generated for this study are available on request to the corresponding author.

## Author Contributions

RM designed the study. AM performed the generation of the required constructs and fungal transformation. NM conducted other described assays in this study and wrote the manuscript with substantial input from RM, AM, MR, and EM. RM and GK coordinated the project. All authors contributed to the article and approved the submitted version.

## Conflict of Interest

The authors declare that the research was conducted in the absence of any commercial or financial relationships that could be construed as a potential conflict of interest.
