# Supplementary material for: MADS-Box Transcription Factor ZtRlm1 Is Responsible for Virulence and Development of the Fungal Wheat Pathogen Zymoseptoria tritici
Source: Front Microbiol. 2020 Aug 18;11:1976. doi: 10.3389/fmicb.2020.01976 (PMC7461931; doi:10.3389/fmicb.2020.01976)
Supplement: Supplementary file 1 [file Table_1.DOCX]

| Supplemental Table 1 Histopathological study to determine and monitor the behavior of ΔZtRlm1 strain during infection stages. | | | | | |
| --- | --- | --- | --- | --- | --- |
| **Strain** | **Total Number** | **Penetrated Number** | **Colonized Number** | **Pycnidia Number** |  |
| ***Ztrlm1#1*** | 10 | 2 | 1 | 0 |  |
| ***Ztrlm1#2*** | 10 | 4 | 3 | 1 |  |
| **WT** | 10 | 10 | 10 | 10 |  |
| **Ectopic** | 10 | 10 | 10 | 10 |  |
