# Supplementary material for: MADS-Box Transcription Factor ZtRlm1 Is Responsible for Virulence and Development of the Fungal Wheat Pathogen Zymoseptoria tritici
Source: Front Microbiol. 2020 Aug 18;11:1976. doi: 10.3389/fmicb.2020.01976 (PMC7461931; doi:10.3389/fmicb.2020.01976)
Supplement: Supplementary file 2 [file Table_2.DOCX]

| Supplemental Table 2 Analysis of variance of branch intensity and hyplahl filaments of strains on PDA and WA media and pycnidia number of strains in 1 cm of infected leaves | | | | | | | | | | |
| --- | --- | --- | --- | --- | --- | --- | --- | --- | --- | --- |
| **Character** | **Source of Variance** | **PDA** | | | |  | **WA** | | | |
|  |  | **df** | **MS** | **F** | **Sig** |  | **df** | **MS** | **F** | **Sig** |
| **Branch intensity** | Treatment | 3 | 4126.8 | 35.7 | .000 |  | 3 | 14515.5 | 40.1 | .000 |
|  | Error | 36 | 115.7 |  |  |  | 36 | 361.8 |  |  |
|  | Total | 39 |  |  |  |  | 39 |  |  |  |
| **Hyphal filaments** | Treatment | 3 | 18597129.9 | 900.2 | .000 |  | 3 | 2822724.3 | 219.5 | .000 |
|  | Error | 36 | 20658.3.1 |  |  |  | 36 | 12855.9 |  |  |
|  | Total | 39 |  |  |  |  | 39 |  |  |  |
| **Pycnidia number in 1 cm** | Treatment | 3 | 84236.9 | 369.3 | .000 |  |  |  |  |  |
|  | Error | 36 | 228.1 |  |  |  |  |  |  |  |
|  | Total | 39 |  |  |  |  |  |  |  |  |
