# Supplementary material for: MADS-Box Transcription Factor ZtRlm1 Is Responsible for Virulence and Development of the Fungal Wheat Pathogen Zymoseptoria tritici
Source: Front Microbiol. 2020 Aug 18;11:1976. doi: 10.3389/fmicb.2020.01976 (PMC7461931; doi:10.3389/fmicb.2020.01976)
Supplement: Supplementary file 3 [file Table_3.DOCX]

| Supplemental Table 3 Dunkan’s Multiple Range Test amonge means of branch intensity and hyplahl filaments of strains on PDA and WA media as well as pycnidia number of strains in 1 cm of infected leaves. | | | | | | | |  |
| --- | --- | --- | --- | --- | --- | --- | --- | --- |
| **PDA** | | | |  | **WA** | |  |  |
| Strains | Number spores | Branch intensity | Hyphal filaments |  | Branch intensity | Hyphal filaments | Pycnidia in 1 cm leaf tip | |
| ***Ztrlm1#1*** | 10 | 6.8**a** | 569.6**a** |  | 12.3**a** | 769.2 **a** | 3.5**a** | |
| ***Ztrlm1#2*** | 10 | 6**a** | 555.5**a** |  | 10.5**a** | 755.5 **a** | 3.1**a** | |
| **WT** | 10 | 43.6**b** | 2939.8**b** |  | 78.7**b** | 1690.8**b** | 165.5**b** | |
| **Ectopic** | 10 | 39.3**b** | 2909.3**b** |  | 76.1**b** | 1674.1**b** | 158.9**b** | |
| Values in the same column followed by the same letters show no significant difference (*p*=0.05). | | | | | | | | |
