# Supplementary material for: MADS-Box Transcription Factor ZtRlm1 Is Responsible for Virulence and Development of the Fungal Wheat Pathogen Zymoseptoria tritici
Source: Front Microbiol. 2020 Aug 18;11:1976. doi: 10.3389/fmicb.2020.01976 (PMC7461931; doi:10.3389/fmicb.2020.01976)
Supplement: Supplementary file 4 [file Data_Sheet_1.DOCX]

Supplemental figure 1 Comparative analysis of the pycnidia formation of the susceptible wheat cv. Obelisk at 20 dpi inoculated by ZtRlm1 mutants and control strains.
